# Positive selection of Kranz and non-Kranz C_4_ phosphoenolpyruvate carboxylase amino acids in Suaedoideae (Chenopodiaceae)

**DOI:** 10.1093/jxb/eru053

**Published:** 2014-03-05

**Authors:** Josh J. Rosnow, Gerald E. Edwards, Eric H. Roalson

**Affiliations:** School of Biological Sciences, Washington State University, Pullman, WA 99164-4236, USA

**Keywords:** *Bienertia*, C_4_ photosynthesis, PAML, phosphoenolpyruvate carboxylase, positive selection analysis, *Suaeda aralocaspica*, Suaedoideae.

## Abstract

In subfamily Suaedoideae, four independent gains of C_4_ photosynthesis are proposed, which includes two parallel origins of Kranz anatomy (sections *Salsina* and *Schoberia*) and two independent origins of single-cell C_4_ anatomy (*Bienertia* and *Suaeda aralocaspica*). Additional phylogenetic support for this hypothesis was generated from sequence data of the C-terminal portion of the phosphoenolpyruvate carboxylase (PEPC) gene used in C_4_ photosynthesis (*ppc-1*) in combination with previous sequence data. *ppc-1* sequence was generated for 20 species in Suaedoideae and two outgroup *Salsola* species that included all types of C_4_ anatomies as well as two types of C_3_ anatomies. A branch-site test for positively selected codons was performed using the software package PAML. From labelling of the four branches where C_4_ is hypothesized to have developed (foreground branches), residue 733 (maize numbering) was identified to be under positive selection with a posterior probability >0.99 and residue 868 at the >0.95 interval using Bayes empirical Bayes (BEB). When labelling all the branches within C_4_ clades, the branch-site test identified 13 codons to be under selection with a posterior probability >0.95 by BEB; this is discussed considering current information on functional residues. The signature C_4_ substitution of an alanine for a serine at position 780 in the C-terminal end (which is considered a major determinant of affinity for PEP) was only found in four of the C_4_ species sampled, while eight of the C_4_ species and all the C_3_ species have an alanine residue; indicating that this substitution is not a requirement for C_4_ function.

## Summary

Phylogenetic analysis of independent gains of C_4_ photosynthesis in Suaedoideae shows differences compared with other C_4_ lineages in amino acids of phosphoenolpyruvate carboxylase under positive selection and their position relative to functional residues.

## Introduction

Phosphoenolpyruvate carboxylase (PEPC) (EC 4.1.1.31) plays an important biochemical role in higher plants by converting bicarbonate (HCO_3_^–^) and phosphoenolpyruvate (PEP), in the presence of Mg^2+^ or Mn^2+^, into the four-carbon acid oxaloacetate (OAA) and Pi (O’Leary, 1982; Chollet *et al.*, 1996; Izui *et al.*, 2004). OAA is readily reduced into the more stable product malate or transaminated to aspartate. Plants that have high levels of PEPC protein in their leaves to generate a pool of aspartate or malate as intermediate products of photosynthesis are known as C_4_ or CAM (Crassulacean acid metabolism) species, as opposed to C_3_ species that use PEPC primarily in an anaplerotic role. C_4_ and CAM plants subsequently de-carboxylate the pool of C_4_ acids, distally or temporally, respectively, to increase the concentration of CO_2_ around Rubisco. Thus, all plant genomes encode several paralogues of PEPC, with only one orthologue being used in the C_4_ pathway (Hermans and Westhoff, 1990; Lepiniec *et al.*, 1994; Chollet *et al.*, 1996; Svensson *et al.*, 2003; Gowik *et al.*, 2006; Rao *et al.*, 2006; Christin *et al.*, 2007, 2011; Besnard *et al.*, 2009). The function of non-C_4_ PEPC paralogues has been summarized recently (O’Leary *et al.*, 2011); they have a role in many plant functions, such as the regulation of stomatal movement, seed development, seed germination, root excretion for abiotic stress acclimation, energy production, carbon storage, nitrogen fixation, and an anaplerotic role in the citric acid cycle. Given the physiological importance of PEPC for C_4_ photosynthesis, numerous studies have shown that PEPC used for C_4_ biochemistry has distinct kinetic differences in comparison with orthologous genes (Ting and Osmond, 1973; Blasing *et al.*, 2000, 2002; Gowik *et al.*, 2006; Lara *et al.*, 2006; Jacobs *et al.*, 2008; Rao *et al.*, 2008). The exact amino acid residues that are responsible for the various observed kinetic difference is still being resolved in order to explain further how PEPC kinetics impact the flux of CO_2_ assimilation through the C_4_ pathway under a wide range of changing conditions.

PEPC in vascular plants is functional as a homodimer of dimers composed of subunits with a molecular mass of 95–116kDa, and is allosterically regulated by the metabolic context of the enzyme (Kai *et al.*, 2003). At physiological pH, PEPC is activated by it substrate Mg-PEP (Tovar-Mendez *et al.*, 1998), phosphorylated sugars such as glucose 6-phosphate (G6P) (Coombs *et al.*, 1973; Wong and Davies, 1973), and neutral amino acids such as glycine, alanine, and serine (Nishikido and Takanashi, 1973; Bandarian *et al.*, 1992; Tovar-Mendez *et al.*, 2000). Dicarboxylic acids such as the downstream products malate and aspartate negatively inhibit PEPC activity (Huber and Edwards, 1975). Enzyme activity can also be modified by phosphorylation on a conserved N-terminal serine residue, which causes a decrease in affinity for dicarboxylic acids and an increase in affinity for PEP (Jiao and Chollet, 1988; Tovar-Mendez *et al.*, 1998). A protein crystal structure for PEPC from *Escherichia coli*, *Zea mays* (C_4_), *Flaveria pringlei* (C_3_), and *F. trinervia* (C_4_) has been resolved to help facilitate understanding of the relationship of amino acid substitutions to PEPC kinetics (Kai *et al.*, 1999; Matsumura *et al.*, 2002; Paulus *et al.*, 2013). Identifying amino acids in the PEPC protein that are under the most selective pressure after being recruited for use in C_4_ biochemistry will further elucidate what changes can potentially enhance C_4_ photosynthesis, potential metabolic limitations, and the regulatory network underlying plant adaptation.

The appearance of C_4_ biochemistry has occurred at least 62 independent times in angiosperms (36 lineages in eudicots, six in the sedges, and 18 in the grasses), making it one of the most common convergent processes studied to date (Sage *et al.*, 2011). Among dicot families, the Chenopodiaceae has the largest number of C_4_ species with the greatest diversity in leaf anatomy, with Kranz anatomy and single-cell C_4_ species as well as C_3_ species (Kadereit *et al.*, 2003; Edwards and Voznesenskaya, 2011). All of the Chenopodiaceae C_4_ genera except *Atriplex* are in the Salicorniodeae*/*Suaedoideae*/*Salsoloideae/Camphorosmoideae (Kadereit *et al.*, 2003; Kadereit and Freitag, 2011). The three different types of C_3_ leaf anatomy within the genus *Suaeda* are characterized according to their sections: *Brezia*, *Vera*, and *Schanginia* (Schütze *et al.*, 2003). There are hypothesized to be four independent origins of C_4_ photosynthesis within the Suaedoideae, including two separate origins of distinctive Kranz C_4_ anatomies, in *Suaeda* sections *Salsina sensu lato* (*s.l.*) and *Schoberia*, and two independent origins of unique single-cell C_4_ anatomy, in *Bienertia* and in *Suaeda aralocaspica* (Kapralov *et al.*, 2006).

Nomenclature for PEPC in higher plants is varied throughout the literature, an artifact that is attributable to the numerous independent characterizations of PEPC genes, independent gene and genome duplication events that occurred after species divergence, as well as non-standardized nomenclature. In the grasses, there are four PEPC genes that have been predominantly characterized, with the PEPC gene that is most often recruited for use in C_4_ photosynthesis being named initially *ppc-C*_*4*_ and subsequently *ppc-B2* (Christin *et al.*, 2007). In the sedges, there are five PEPC genes that have been predominantly characterized, with the gene being recruited for use in C_4_ being labelled *ppc-1* (Besnard *et al.*, 2009). In *Arabidopsis* there are four PEPC genes that were arbitrarily numbered 1–4 (Sanchez and Cejudo, 2003). One of the earliest dicot C_4_ PEPC genes analysed was in *Flaveria* (Asteraceae), where three genes were identified and labelled A, B, and C, with the *ppc-A* gene being identified as the gene recruited for use in the C_4_ photosyntehic pathway (Hermans and Westhoff, 1990; Engelmann *et al.*, 2003). Alphabetical nomenclature for PEPC genes was subsequently used in *Alternthera* (Amaranthaceae) where the three characterized PEPC genes were phylogenetically sister to the *Flaveria* PEPC genes (Gowik *et al.*, 2006). More recent phylogenetic analysis shows that there are two paralogous eudicot PEPC genes, with the one most often being recruited for use in the C_4_ photosynthetic pathway being labelled *ppc-1*, with the exception of *Flaveria* where the apparent loss of the *ppc-1* gene required the recruitment of a twice duplicated *ppc-2* gene (Christin *et al.*, 2011). Here the nomenclature of Christin *et al.* (2011) is followed, since this is to date the most detailed phylogenetic study of eudicot PEPC genes, even though only two of the 3–5 eudicot PEPC genes are presented.

Genes from closely related species tend to have a high amino acid homology, and in the case of PEPC, ~10% (or ~100 residues) are invariant across all PEPC genes (Nakamura *et al.*, 1995). Orthologous protein variation is the result of changes to the nucleotide sequence that alter the codon and resulting amino acid. Changes to the coding sequence of a gene are often classified as being either a non-synonmous (dN) substitution that alters the resulting amino acid, or a synonymous (dS) substitution that changes the codon but does not change the amino acid. The direction of selective pressure at each amino acid residue is determined by comparing the rates of dN and dS substitutions across orthologous proteins, usually expressed as the ratio of dN to dS (ω=dN/dS). Equal rates of both types of substitutions (ω=1) suggest neutral evolution or low selective pressure for a specific amino acid at that residue. A low number of dN substitutions relative to the number of dS substitutions (ω<1) indicates purifying selection pressure against changes to the amino acid present. A high number of dN substitutions relative to dS substitutions (ω>1) suggests that the new amino acid present offers some fitness advantage probably associated with adaptive change (Yang and Nielsen, 2002; Huelsenbeck and Dyer, 2004). At the molecular level, there are functional constraints across the protein, so the type of selective pressure at each residue is generally purifying. In most proteins, a functional difference is often the result of positive selection at only a few sites (Yang *et al.*, 2005; Christin *et al.*, 2008; Kapralov *et al.*, 2012). Phylogenetic analysis in the grasses showed that *ppc-B2* was recruited eight independent times for use in the C_4_ pathway (Christin *et al.*, 2007). During this switch to C_4_, 21 amino acids evolved under positive selection and converged to similar or identical amino acids, some of which have also been recorded in non-grass C_4_ species (Blasing *et al.*, 2000; Gowik *et al.*, 2006; Christin *et al.*, 2007, 2011; Besnard *et al.*, 2009). Such convergence at some sites, such as serine at position 780, appears to reflect the need for specific amino acid residues for C_4_ function, whereas at other sites there appears to be a requirement for loss of the C_3_-associated amino acid. These substitutions are thought to optimize PEPC for C_4_ photosynthetic function.

The specific aims of this study are to: (i) find additional phylogenetic support for the four origins of C_4_ photosynthesis in Suaedoideae by using PEPC sequence data; (ii) identify which PEPC paralogous gene is being recruited for use in C_4_ photosynthesis in comparison with other previously characterized PEPC genes; (iii) identify any amino acids under positive selection in the PEPC gene used in C_4_ photosynthesis; and (iv) determine the spatial location of positively selected amino acids in relation to catalytic and allosteric regulatory amino acids.

## Materials and methods

### Plant material

All plants used in this study were started from seed and were grown in controlled environmental chambers (Econair GC-16; Bio Chambers). Seedlings were started under lower light [100 photosynthetic photon flux density (PPFD; μmol quanta m^–2^ s^–1^)] and temperature conditions with a day/night temperature of 25/22 °C and a photoperiod of 14/10h, and then moved to high light and temperature conditions (1000 PPFD, with a day/night temperature of 35/25 °C and a photoperiod of 14/10h) once plants were well established. A few leaves from 2- to 6-month-old plants were used for DNA extraction.

### DNA sequencing

The PEPC gene *ppc-1* was sequenced for two *Bienertia* species and 18 *Suaeda* species, with two *Salsola* species sequenced as outgroups for the selection analyses. The PEPC gene *ppc-2* was sequenced for 12 *Suaeda* species. DNA was extracted from 250mg of plant material using the CTAB (cetyltrimethylammonium bromide) method following the protocol of Doyle and Doyle (1987). Primers were developed to similar regions previously analysed for positive selection in order to amplify exons 8–10 (Supplementary Table S1 available at *JXB* online). Initial PCR conditions were 2min at 95 °C, followed by 35 cycles of: 30 s at 95 °C, a 30 s 52 °C annealing step, and a 3min extension at 72 °C. The PCR product was visualized and purified using a PCR clean-up kit according to the manufacturer’s protocol (Qiagen, USA). Purified PCR product was cloned into pGEM T-easy vector using the manufacturer’s protocol (Promega, USA). Single colonies were grown overnight and plasmid DNA was purified using alkaline lysis with SDS (Sambrook and Russell, 2001). Plasmid inserts were PCR amplified using GOTaq (Promega, USA), and Sp6 and T7 primers, and were visualized on a gel. Prior to sequencing, the PCR product was mixed with 2.5U of antarctic phosphatase and 4U of exo-sap nuclease in antarctic phosphatase buffer (New England BioSciences, USA) to degrade primers and nucleotides, and subsequently diluted 1:10. Sequencing reactions were performed using the Big Dye terminator master mix v3.1 (Applied BioSciences, USA), using sequence-specific internal primers along with Sp6 and T7 (Supplementary Table S1). Sequencing was carried out by Operon Sequences (USA) and at Washington State University genomics core. Sequence data were assembled using Sequencher software (USA). Nucleotide sequences were translated, aligned, and visualized using Se-Al and MacVector (USA). All sequences were deposited in GenBank (Supplementary Table S2).

### Phylogenetic analyses

Three DNA sequence data matrices were analysed in this study: (i) the previously published matrix of the nuclear ribosomal internal transcribed spacer (ITS), along with the chloroplast intergenic spacers of *atpB*-*rbcL*, and *psbB*-*psbH* from Kapralov *et al.* (2006) (Supplementary Table S3 at *JXB* online), combined with the third nucleotide from each codon and introns for *ppc-1* from the subsample of the species sampled; (ii) *ppc-1* and *ppc-2* coding sequence from Suaedoideae sequenced here and other eudicot sequences from GenBank; and (ii) *ppc-1* third position sites and introns of the suaedoids sequenced in this study with two *ppc-1 Salsola* samples used as the outgroup. Alignments of PEPC genes and their introns were conducted with MUSCLE (Edgar, 2004), and visually inspected using Se-Al. Maximum likelihood (ML) analyses were performed using the GTRGAMMA model in RAxML version 7.2.8 (Stamatakis, 2006; Silvestro and Michalak, 2012) with 1000 bootstraps using multithreading on four cores.

### Positive selection analysis

Positive selection analysis used the ML tree from the *ppc-1* suaedoid data set analysis described above, consisting of eight C_3_ species, nine C_4_ Kranz species, and three single-cell C_4_ species, of which *Bienertia sinuspersici* had two *ppc-1* accessions. Two *Salsola* species (one C_3_ and one C_3_–C_4_ intermediate) were used as outgroups. To test for positive selection at particular sites of the *ppc-1* gene, the *codeml* program in the PAML v4.4 package was used to perform likelihood ratio tests (LRTs) to identify the best model for codon change while concurrently identifying dN amino acid changes that are under positive selection (Yang, 2007). Each model adds additional parameters to try and fit the data better by assuming similar ω values either across all of the phylogeny (site test) or on pre-specified C_4_ branches only (branch-site test). A comparison of LRT scores shows which model fits the data the best. Details of each model are as follows. Model M0 allows for a single ω value across the whole phylogenetic tree at all sites. Subsequent models allow ω to vary at different sites. Model M1a (nearly neutral) allows for two rates of ω to vary between 0 and 1, while Model M2a (positive selection) is the same as Model M1a but allows for an additional rate of ω to be >1. Model M8a assumes a discrete beta distribution for ω, which is constrained between 0 and 1 including a class with ω=1, similar to Model M8 which allow the same distribution as M8a but has an extra class under positive selection with ω>1. Branch-site tests, using pre-specified branches where changes associated with C_4_ photosynthesis are hypothesized to have occurred (foreground branches), were made with the null Model A1. This allows ω ratios to vary among sites and among lineages, and it also provides two additional classes of codons with ω=1 along pre-specified foreground branches, while restricting ω to be ≤1, on background branches. The alternative Model A allows ω to vary between 0 and 1, be equal to 1 for all branches, and also has two additional classes of codons under positive selection with ω>1 along pre-specified foreground branches while restricting ω to either 0–1 or ω=1 on background branches. C_4_ lineages were marked as foreground branches.

For all LRTs, the null model is a simplified version of the selection model, with fewer parameters, and is thus expected to provide a poorer fit to the data (lower maximum likelihood). The null models (M1a, M8a, and A1) do not allow codons with ω>1, whereas the selection models (M2a, M8, and A) are alternative models that allow for codons with ω>1. The significance of the LRTs was calculated assuming that twice the difference in the log of maximum likelihood between the two models was distributed as a χ^2^ distribution with the degrees of freedom (df) given by the difference in the number of parameters in the two types of models (Yang and Nielsen, 2002; Yang and Swanson, 2002). For the M1a–M2a comparison df=2, and for M8a–M8 and A1–A comparisons df=1. Each LRT was run at least twice using different initial omega values to test for suboptimal local peaks. To identify amino acid sites potentially under positive selection, the parameter estimates from M2a, M8, and A models were used to calculate the posterior probabilities that an amino acid belongs to a class with ω>1, using the Bayes empirical Bayes (BEB) approaches implemented in PAML (Yang *et al.*, 2005).

### Structural analysis of PEPC

The recently published PEPC (*ppc-2*) protein structure from the C_4_ species *F. trinervia* (Asteraceae) (Paulus *et al.*, 2013) was obtained from the RCSB Protein data bank (www.rcsb.org, 3ZGB). Throughout this paper, the numbering of PEPC residues is based on the maize *ppc-B2* sequence CAA33317 (Besnard *et al.*, 2003) for easy comparison with previous studies. The properties, locations, and spatial relationships of individual amino acids in the PEPC structure were analysed using CUPSAT and PyMOL (Schrödinger; Parthiban *et al.*, 2006). Figures of the atomic structures and distances measurements were made with PyMOL, and formatted using Adobe Photoshop CS5 (USA).

## Results

### Phylogenetic analyses

**Fig. 1.**
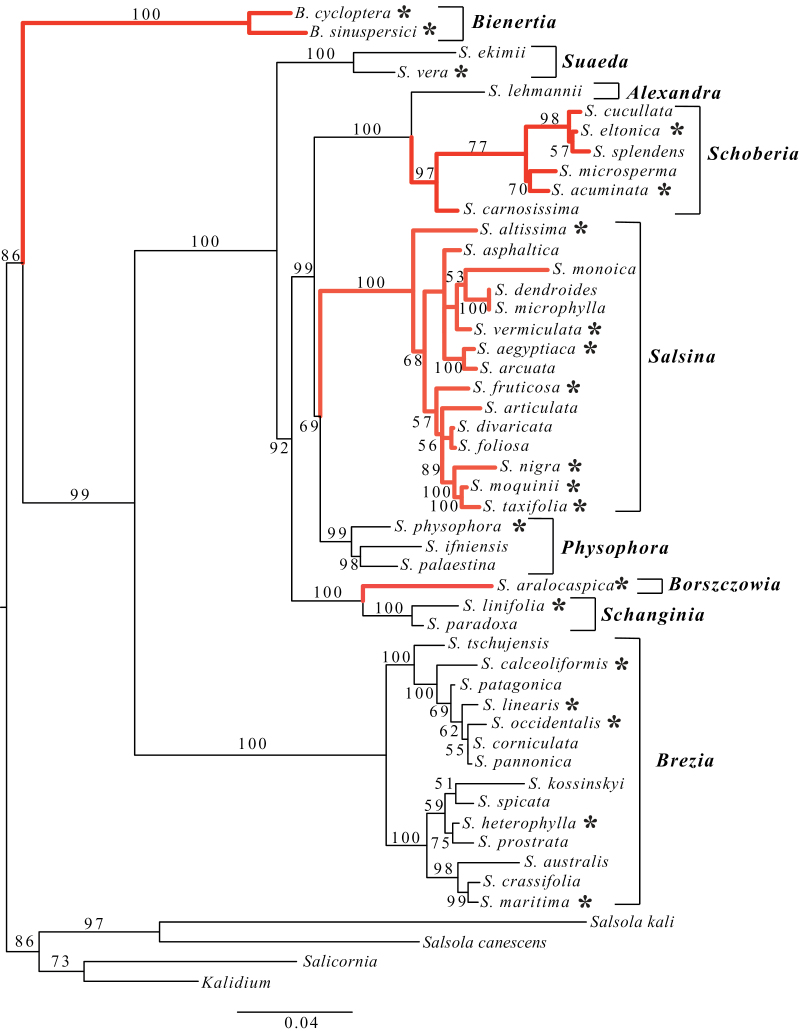
Suaedoideae phylogeny using ITS, *atp-rbcL*, *psbB-psbH*, and *ppc1* third position plus intron sequence. Number above branches refer to bootstrap percentages. Clades leading to C_4_ photosynthesis are highlighted in red. Taxa used for positive selection analysis are indicated with an asterisk. Abbreviations: *B*., *Bienertia*; *S*., *Suaeda*. Bracketed names refer to section names within Suaedoideae.

Analysis of third position sites and introns of *ppc-1*, in combination with previous ITS, *atpB*-*rbcL*, and the *psbB*-*H* sequence data, provided additional support for relationships in Suaedoideae showing four independent origins of C_4_. The ML phylogenetic tree (Fig. 1) for 46 Suaedoideae species and four outgroup species is in agreement with previously obtained phylogenies for this subfamily (Schütze *et al.*, 2003; Kapralov *et al.*, 2006). Previous phylogenetic analyses of relationships within *Suaeda* provided a strongly supported phylogenetic hypothesis of relationships in the clade, with the exception of two branches: the branch grouping *Schoberia*+*Alexandra* to *Physophora* and the branch grouping *Schanginia*+*Borszczowia* to *Suaeda* both had bootstrap support <50% (Kapralov *et al.*, 2006). With the addition of *ppc-1* data to the previously analyzed data sets, a similar but generally much more strongly supported phylogeny suggests a grade of *Suaeda*, *Schanginia*+*Borszczowia*, *Schoberia*+*Alexandra*, *Physophora*, and *Salsina* clades (Fig. 1). Among the clades, the only branch grouping major clades that does not have >90% bootstrap support is the *Physophora*+*Salsina* branch (bootstrap support=69%). Despite this, these results continue to support strongly four independent gains of C_4_ photosynthesis within the Suaedoideae, including two parallel origins of distinctive Kranz C_4_ anatomy in *Suaeda* sections *Salsina s.l.* and *Schoberia*, and two independent origins of unique single-cell C_4_ anatomy in *Bienertia* and *Suaeda aralocaspica*.

**Fig. 2.**
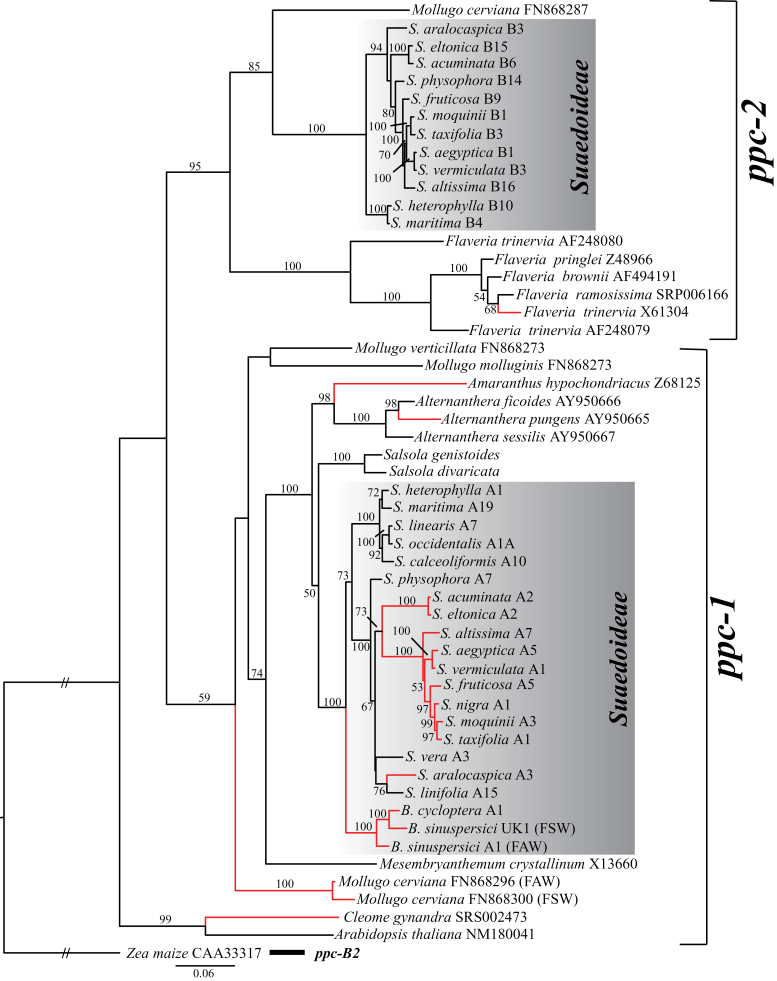
Eudicot PEPC exon 8, 9, and 10 phylogeny rooted on the monocot *ppc-B2* maize gene. Accessions numbers are indicated after species names for sequences retrieved from GenBank. Paralogous PEPC genes (*ppc-1* and *ppc-2*) are delimited on the right. Eudicot families are indicated on the right. Branches leading to C_4_ clades are in red. Numbers above branches are the bootstrap percentages.

The phylogenetic relationships of eudicot PEPC genes, using exons 8, 9, and 10, rooted on the monocot *ppc-B2* maize gene, shows that the *ppc-1* gene that was recruited for use in C_4_ photosynthesis in Suaedoideae is the same orthologous gene that has been previously shown to be recruited for use in C_4_ photosynthesis in other C_4_ eudicot species (Fig. 2). The exception to this parallel recruitment of the same PEPC gene is found in the Asteraceae where the paralogous *ppc-2* gene is recruited for use in C_4_ photosynthesis. There is strong bootstrap support for all Suaedoideae *ppc-1* genes being sister to closely related Amaranthaceae *ppc-1* genes.

### Positive selection analyses of the PEPC coding sequence

The sequenced region of *ppc-1* and *ppc-2* includes exons 8, 9, and 10 and accounts for 511 of the 973 (53%) amino acids present in the PEPC protein. Comparison of the *ppc-1* gene across the 22 species showed that 372 of these 511 (73%) amino acids are conserved. The same sequenced region for the *ppc-2* gene, for 12 of the 22 sampled species, showed that 449 of the 506 (88%) amino acids are conserved.

A phylogenetic tree, generated from intron and third position sites, was used to obtain a supported tree that is minimally affected by selective pressures (Supplementary Fig. S1 at *JXB* online). The tree had 23 tips, which included 22 sampled species and an additional tip for the variation found at residue 780 in the *B. sinuspersici ppc-1* gene (denoted FAW for an alanine residue, or FSW for a serine residue present at position 780). Identification of codons under positive selection was performed using the software package PAML, which provided a likelihood score for each model, that was subsequently used to test for significance between the null and selection model. LRT showed that the site models assuming positive selection (M2a and M8) did not fit the data better than models without positive selection (M1a and M8a), with a *P*-value of 0.5273 and 0.0192, respectively (Table 1).

**Table 1.** Comparison of modelled amino acid model change in the Suaedoideae ppc-1 gene, to identify sites under positive selection

Model with positive selection^*a*^				Null model^*a*^			LRT^*b*^	*P*-value
Model	Log-likelihood	Parameters^*c*^	Positively selected sites^*d*^	Model	Log-likelihood	Parameters^*c*^	2L	
Analysis for positively selected sites common for C_3_ and C_4_ clades								
M2a	–6741.7	κ=2.94, p_0_=0.82, ω_0_=0.07, p_s_=0.003, ω_S_=3.24	None	M1a	–6742.34	κ=2.92, p_0_=0.82, ω_0_=0.07	1.28	0.5273
M8	–6740.28	κ=2.89, p_0_=0.98, p=0.24, q=1.0, ω_S_=1.93	None	M8a	–6743.02	κ=2.86, p_0_=0.96, p=0.19, q=0.7	5.48	0.0192
Analysis for positive selection along branches leading to C_4_ clades								
A	–6729.78	κ=2.95, p_0_=0.81, ω_0_=0.08, p_s_=0.04, ω_S_=4.2	***733***, 868	A1	–6737.24	κ=2.89, p_0_=0.77, ω_0_=0.06	14.9	0.0001
Analysis for positive selection along branches leading to Kranz C_4_ clades								
A	–6732.25	κ=2.94, p_0_=0.77,ω_0_=0.06, p_s_=0.06, ω_S_=3.64	485, 519, 735	A1	–6735.67	κ=2.91, p_0_=0.71, ω_0_=0.06	6.8	0.0091
Analysis for positive selection along branches leading to single-cell C_4_ clades								
A	–6742.34	κ=2.92, p_0_=0.82, ω_0_=0.07, p_s_=0.00, ω_S_=NA	None	A1	–6742.34	κ=2.92, p_0_=0.82, ω_0_=0.07	0	1
Analysis for positive selection along all C_4_ branches								
A	–6702.46	κ=2.91, p_0_=0.74, ω_0_=0.05, p_s_=0.18, ω_S_=1.5	480, 513, 627, 662, 695, 707, 733, 744, 794, 863, 868, 880, 931	A1	–6705.34	κ=2.86, p_0_=0.69, ω_0_=0.04	5.08	0.0242

^*a*^ M1a (nearly neutral), M2a (positive selection), M8a (beta and ω=1), and M8 (beta and ω) are PAML site models; A1 and A are PAML branch-site models.

^*b*^ LRT is the likelihood ratio test; 2L is twice the difference of model log-likelihoods.

^*c*^ κ is the transition/transversion rate ratio; ω is the dN/dS ratio; ω_s_ is the dN/dS ratio in a class under putative positive selection; p_0_ and p_s_ are the proportion of codons with ω<1 and ω>1, respectively; p and q are parameters of beta distribution in the range (0, 1).

^*d*^ Sites listed are those at which positive selection is detected at the significance level of >95%, or >99% in bold italics.

To test if codon selection occurs specifically in C_4_ clades, the branch site Model A, which allows for ω>1 along foreground branches (branches where C_4_-specific changes were hypothesized to occur), was compared with the null Model A1, which only allows for ω≤1 along foreground and background branches. This model comparison was made by labelling foreground branches in four different ways: (i) only those foreground branches leading to C_4_ clades; (ii) only those foreground branches leading to Kranz C_4_ clades; (iii) only those branches leading to single-cell C_4_ clades; or (iv) by labelling all branches within C_4_ clades. Comparing the selection Model A with the null Model A1 showed that labelling of just the branches leading to C_4_ clades was the most significant, with a *P*-value <0.0001 (Table 1). Labelling just the branches leading to Kranz C_4_ clades, as well as all branches within all C_4_ clades, both produced significant results; *P*-value 0.0091 and 0.0242, respectively (Table 1). There was no variation in the likelihood score for the selection model compared with the null model when labelling just foreground branches leading to single-cell C_4_ clades (Table 1). There was some variation in sites identified under selection depending on how the foreground branches were labelled.

### Sites under positive selection

There were no codons identified as being under positive selection with a posterior probability >0.95 by BEB in the M2A or M8 model (Table 1). There were two codons (position 733 and 868) that were shown to be under positive selection with a posterior probability >0.95 by BEB when only branches leading to C_4_ clades were labelled as foreground branches, with position 733 being the only residue identified to have a posterior probability >0.99 by BEB in Model A (Table 1). Both sites had four alternative amino acids present in this data set, an amino acid present mostly in C_3_ species, and one of three amino acids present in C_4_ species (Table 2). Only 733 had a substitution present in all the C_4_ species sampled from the C_3_ amino acid, while 868 had a substitution in all C_4_ species sampled except the two *Bienertia* species. Model A identified three codons (485, 519, and 735) that were shown to be under positive selection with a posterior probability >0.95 by BEB, when only branches leading to Kranz C_4_ clades were labelled as foreground branches (Table 1). Substitutions at codons 485 and 735 had two possible amino acids present in the data set, and all species in the *Salsina* clade had a substitution to the C_4_ amino acid (Table 2; Supplementary Table S4 at *JXB* online). Additionally, codon 519 also had two amino acids present in the data set, and a substitution to the C_4_ amino acid was only present in the two *Schloberia* sampled species (Table 2; Supplementary Table S4). There were no codons identified as being under positive selection that was specific to branches leading to single-cell C_4_ clades (Table 1). By labelling all C_4_ branches as foreground branches, 13 codons were identified as being under positive selection (480, 513, 627, 662, 695, 707, 733, 744, 794, 863, 868, 880, and 931) with a posterior probability >0.95 by BEB (Table 1). Two of these residues (733 and 868) were identified as being on branches leading to C_4_ clades.

**Table 2.** Characteristics of amino acid replacements under positive selection in the C_4_ lineages of Suaedoideae

AA no.^*a*^	AA change C_3_→C_4_	Type of change^*b*^	ΔH^*c*^	ΔP^*d*^	ΔV^*e*^	SA^*f*^ (%)	ΔG^*g*^ (kJ mol^–1^)	No. of C_3_/ no. of C_4_^*h*^	No. of transitions for C_4_ AA	No. of transversions for C_4_ AA	Location of residue
480	D→E	D→D	0.0	–0.7	27.3	92.7	S (0.15)	0/11	–	1	α-Helix 18
485^*i*^	K→A	R→A	5.7	–3.2	–80.0	40.3	DS (–1.12)	0/7	1	1	α-Helix 19
513	D→A	D→A	5.3	–4.9	–22.5	18.7	DS (–1.17)	0/9	–	1	α-Helix 20
519^*j*^	H→K	R→R	–0.7	0.9	15.4	46.7	S (2.04)	0/2	–	2	α-Helix 20
627	V→I	A→A	0.3	–0.7	26.7	10.3	S (1.4)	0/9	1	–	α-Helix 25
662	D→E	D→D	0.0	–0.7	27.3	45.1	DS (–1.11)	0/3	–	1	Loop
695	T→V	P→A	4.9	–2.7	23.9	0.7	DS (–4.39)	0/1	2	–	α-Helix 28
	T→I	P→A	5.2	–3.4	50.6	0.7	DS (–0.97)	0/1	1		α-Helix 28
707	I→M	A→A	–2.6	0.5	–3.8	47.6	DS (–0.64)	0/1	1	–	Loop
	I→L	A→A	–0.7	–0.3	0.0	47.6	DS (–1.55)	0/2	–	1	Loop
	I→S	A→P	–5.3	4.0	–77.7	47.6	S (0.38)	0/1	1	–	Loop
	I→T	A→P	–5.2	3.4	–50.6	47.6	DS (–0.3)	0/1	1	–	Loop
733	F→M	F→A	–0.9	0.5	–27.0	39.9	DS (–3.73)	0/2	1	1	Loop
	F→L	F→A	1.0	–0.3	–23.2	39.9	DS (–3.14)	0/8	–	1	Loop
	F→R	F→R	–7.3	5.3	–16.5	39.9	DS (–2.42)	0/2	1	1	Loop
735^*i*^	E→N	D→P	0.0	–0.7	–24.3	48.7	DS (–0.76)	0/7	1	1	Loop
744	L→C	A→P	–1.3	0.6	–58.2	29.5	DS (–2.63)	0/2	–	2	α-Helix 30
	L→R	A→R	–8.3	5.6	6.7	29.5	DS (–4.14)	0/2	–	1	α-Helix 30
780	A→S	A→P	–2.6	1.1	0.4	0.0	DS (–3.1)	0/4	–	1	α-Helix 32
794	F→I	F→A	1.7	0.0	–23.2	0.0	DS (–2.03)	0/6	–	1	α-Helix 34
	F→M	F→A	–0.9	0.5	–27.0	0.0	DS (–2.51)	0/2	1	1	α-Helix 34
	F→L	F→A	1.0	–0.3	–23.2	0.0	DS (–0.87)	0/1	–	1	α-Helix 34
863	S→K	A→R	0.6	2.1	79.6	15.7	DS (–0.22)	0/1	1	1	α-Helix 38
	S→D	A→D	–2.7	3.8	22.1	15.7	S (0.07)	0/1	2	–	α-Helix 38
	S→N	A→P	–2.7	2.4	5.1	15.7	S (2.2)	0/9	1	–	α-Helix 38
	S→T	P→P	0.1	–0.6	27.1		DS (–0.43)	0/1	–	1	α-Helix 38
868	K→R	R→R	–0.6	–0.8	4.8	16.5	DS (–0.58)	0/2	1	–	α-Helix 38
	K→Q	R→P	0.4	–0.8	–24.8	16.5	DS (–0.07)	0/1	–	1	α-Helix 38
	K→L	R→A	7.7	–3.4	–1.9	16.5	DS (–0.88)	0/7	–	2	α-Helix 38
880	D→N	D→P	0.0	–1.4	3.0	46.5	DS (–1.49)	0/5	1	–	Loop
	D→E	D→D	0.0	–0.7	27.3	46.5	DS (–0.22)	0/1	–	1	Loop
	D→Y	D→A	2.2	–6.8	82.5	46.5	DS (–3.79)	0/1	–	1	Loop
931	M→I	A→A	2.6	–0.5	3.8	–	–	0/2	1	–	α-Helix 39

^*a*^ Amino acid (AA) numbering is based on the maize sequence after Hudspeth and Grula (1989).

^*b*^ Side chain type changes: A, non-polar aliphatic; P, polar uncharged; D, polar negatively charged; R, polar positively charged.

^*c*^ Hydropathicity difference (Kyte and Doolittle, 1982).

^*d*^ Polarity difference (Grantham, 1974).

^*e*^ van der Waals volume difference (Zamyatin, 1972).

^f^ Solvent accessibility calculated using the *Flaveria trinervia ppc-2* structure (pdb file 3ZGE) by CUPSAT (Parthiban *et al*. 2006).

^*g*^ Overall stability of the protein predicted using the *F. trinervia ppc-2* structure (pdb file 3ZGE) by CUPSAT (Parthiban *et al.*, 2006): DS, destabilizing; S, stabilizing.

^*h*^ Number of C_3_ or C_4_ Suaedoideae species that have the indicated amino acid substitution (amino acid on right side of arrow).

^*i*^ Specific for Salsina Kranz anatomy.

^*j*^ Specific for Schoberia Kranz anatomy.

The amount of substitution at a particular residue for a C_4_ amino acid from a C_3_ amino acid varies quite considerably across the data set. Residues which have a low frequency of substitutions are found at positions 695 and 931 where only the two *Bienertia* species have a difference in the amino acid present, while at positions 662 and 744 only three or four Kranz C_4_ species, respectively, have a different amino acid present (Table 2; Supplementary Table S4 at *JXB* online). Residues that have a high frequency of substitutions, but not in all species, are found at positions 480, 513, 627, 794, and 863 (Table 2; Supplementary Table S4). Additionally, sites under selection show differences in the degree of parallelisms. For example, residues at positions 480, 485, 513, 627, and 735 all changed to an identical amino acid along different *ppc-1* lineages, while residues at positions 707, 733, 794, 863, 868, and 880 changed to different amino acids (Table 2). This suggests that the C_4_ characteristics might be conferred by a change to a specific amino acid or by the absence of a particular amino acid.

**Fig. 3.**
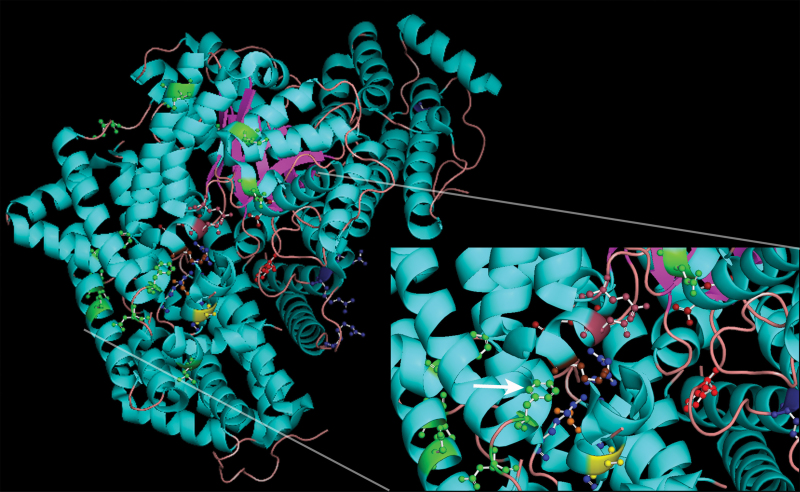
Cartoon representation of the C_4_ PEPC enzyme structure of *Flaveria trinervia* (*ppc-2* gene) (Paulus *et al.*, 2013), showing the location of functional residues in comparison with sites that were identified to be under positive selection in Suaedoideae. Green residues are sites under positive selection, biochemically essential residues are in a shade of red (histone 177 is bright red, Mg^2+^-binding sites are ruby, PEP- and HCO_3_^–^-binding sites are dark red, Lys606 is brown), allosteric regulatory sites are in a shade of blue (deep-blue residues are glucose 6-phosphate-binding sites, light blue are malate/aspartate-binding sites), Gly890 is orange, and Ser780 is yellow (maize numbering). Residue 733 is indicated with a white arrow. The proposed residue function is adapted from Kai *et al.* (2003).

Spatial analysis of the sites identified to be under positive selection indicates that most identified residues are found around the enzyme reaction site/hydrophobic pocket (Fig. 3). Residues close to the G6P-binding pocket were not analysed in this study since they are present in exons 2, 3, and 4. Some residues (794, 863, 868, and 880) under positive selection are in the vicinity of the allosteric site for aspartate and malate regulation (Fig. 3). Two residues (480 and 485) identified to be under positive selection are far from the reaction site and are most probably involved in the dimer–dimer interaction. Interestingly, no sites involved in the β-barrel structure were identified to be under selection, meaning all sites were located in an α-helix or a connecting loop (Table 2).

## Discussion

### Recruitment of the *ppc-1* gene in four gains of C_4_ photosynthesis in Suaedoideae

Extensive phylogenetic analyses on the relationships of higher plant PEPC genes has been previously carried out in families Cyperaceae, Poaceae, and Molluginaceae to determine which PEPC gene is recruited for use in C_4_ biochemistry (Christin *et al.*, 2007, 2011; Besnard *et al.*, 2009; Christin and Besnard, 2009). In the monocots, the same *ppc-B2* orthologue is recruited for each independent gain of C_4_ photosynthesis, except for *Stipagrostis* where the *ppc-B2* orthologue is lost and *ppc-aL1b* is instead used for C_4_ photosynthesis (Christin and Besnard, 2009). In the core eudicots, there are two primary PEPC gene lineages that have been studied to date: *ppc-1* and *ppc-2* (Fig. 2) (Christin *et al.*, 2011). Results from this study show that C_4_ Suaedoideae species (Chenopodiaceae) all recruit the orthologous *ppc-1* gene for C_4_ use (Fig. 2), as has been found in Amaranthaceae and Molluginaceae (Gowik *et al.*, 2006; Christin *et al.*, 2011). This differs from *Flaveria* (Asteraceae) where the paralogous *ppc-2* gene was recruited (Hermans and Westhoff, 1992; Westhoff and Gowik, 2004).

### Selection for PEPC amino acid residues in C_4_ species

In Suaedoideae, there are four independent *ppc-1* lineages that have a number of non-synonomyous substitutions (Table 1; Fig. 2). This is analogous to previous positive selection analyses on PEPC that showed there are important adaptive changes to the PEPC sequences in C_4_ species (Christin *et al.*, 2007, 2012; Besnard *et al.*, 2009). What is divergent about these changes are the residues at which the changes have occurred. Of the 15 amino acids identified to be under positive selection in this analysis (Table 1), only residue 733 was previously identified to be under selection in both the grasses and sedges, while residues 794 and 863 were identified to be under selection in the grasses (Christin *et al.*, 2007; Besnard *et al.*, 2009). This variation in what amino acids are under selection may be attributed to the recruitment of different PEPC paralogues with different starting amino acid sequences. However, when looking at paralogous PEPC genes that were recruited for use in C_4_ photosynthesis in the grasses, parallel changes for eight of the 21 codons previously shown to be under positive selection in C_4_ grasses (*ppc-B2* gene) also had the same amino acid substitution on the paralogous *ppc-aL1b* branch of the C_4_ *Stipagrostis* (positions 517, 531, 572, 579, 625, 665, 733, and 780) (Christin and Besnard, 2009). The positive selection of *ppc-1* amino acid residues in C_4_ Suaedoideae species suggests that there is some fitness advantage associated with these changes. The lack of parallel substitutions between monocots and Suaedoideae might also be due to structural and ecological differences associated with optimization for C_4_ function. For C_4_ function there needs to be spatial separation between the site of atmospheric CO_2_ capture and the site of decarboxylation in the C_4_ cycle, which provides resistance to leakage and allows CO_2_ to be concentrated and assimilated by Rubisco. There are major differences in anatomy and structural differences in how this is achieved among the four independent origins of C_4_ in Suaedoideae (and in comparison with monocots), namely in the arrangement of the cytoplasmic compartments of the two types of single-cell C_4_ species, and in the position of organelles in bundle sheath cells between the two Kranz lineages (Schütze *et al.*, 2003; Edwards and Voznesenskaya, 2011). These are succulent halophytes living in semi-arid deserts where high temperature, limited water, and saline soils could all contribute to CO_2_ limitations on photosynthesis where C_4_ would be beneficial. The evolution of C_4_ photosynthesis in Chenopodiaceae was promoted by adaptation of species to dry and/or saline habitats (Kadereit *et al.*, 2012). Further kinetic analyses of PEPC in C_4_ lineages are needed to characterize differences that may be linked to optimization for C_4_ function. Thus the exact PEPC amino acid modifications that are necessary for optimized C_4_ biochemical kinetics seem to vary across deeply divergent C_4_ origins.

With respect to PEPC function, there have been several residues which, when mutated individually, are clearly shown to be essential for enzyme catalytic function (reviewed in Kai *et al.*, 2003). However, substitution for amino acids along the PEPC sequence that either has no effect, or actually improves enzyme kinetics for function in C_4_, is hard to determine experimentally. Thus, it is difficult to know if all the observed changes in both Suaedoideae C_4_ *ppc-1* genes and previously analysed PEPC genes in the grasses and sedges are absolutely necessary, or act in a synergistic way, to improve the enzyme function for C_4_ biochemistry. Closely related species that use the same PEPC gene for C_4_ photosynthesis probably have an optimal molecular path for amino acid changes that is different from that of distantly related species that recruited a different PEPC gene (Besnard *et al.*, 2009). With all of the family-specific PEPC adaptive changes that alter PEPC kinetics for use in C_4_ photosynthesis, residue 733 is the only codon that underwent a similar change in the sedges, grasses, and Suaedoideae (Supplementary Table S4 at *JXB* online). There seems to be no requirement for a specific residue at this location, as four different amino acid substitutions are observed in C_4_ species (Table 2; Supplementary Table S4). All of the substitutions at position 733 are from the bulky C_3_ phenylalanine to the less bulky amino acids methionine, leucine, or arginine in Suaedoideae, or, comparatively, a valine substitution in the grasses and sedges. Substitution at 733 in Suaedoideae was observed in all C_4_ species sampled, while none of the C_3_ species had this substitution. This substitution is also observed in some C_4_ species such as *Amaranthus hypochondriacus*, *Cleome gynandra*, and some grasses and sedges, but not all C_4_ species (Supplementary Table S4). Single amino acid substitutions of PEPC have been recently shown to have dramatic effects on enzyme kinetics (Paulus *et al.*, 2013). While no analysis has specifically analysed the effect of a substitution at residue 733, it is in close proximity (>4 Å) to a lysine residue that is conserved across higher plant PEPC genes (606 maize numbering/600 *Flaveria* numbering) (Supplementary Fig. S2 at *JXB* online). Lysine (606/600) is proposed to be involved in substrate binding through mutation analysis that showed that when Lys606 is mutated to an arginine or threonine, the *K*_m_ for HCO_3_^–^ increased up to 9-fold, but there was a minimal effect on the overall maximal velocity (*V*_max_) (Gao and Woo, 1995). The exact function of Lys606 is not known since the residue is not required for enzyme activity, but when mutated becomes less active at physiological pH and is more inhibited by malate (Gao and Woo, 1995). Closer analysis of the phenylalanine substitution at 733 shows that every substitution is to a smaller amino acid (Table 2), that increases the space between Lys606 and changes the solvent-exposed surface (Supplementary Fig. S2). If Lys606 is involved in HCO_3_^–^ or, to a lesser extent, PEP binding, as has been proposed (Kai *et al.*, 2003), then by making it more accessible by substituting out phenylalanine, the *K*_m_ for HCO_3_^–^, and possibly for PEP may increase. Substitution at 733 could thus be beneficial to C_4_ plants, as this could increase the rate of HCO_3_^–^ utilization and potentially C_4_ acid generation, which indicates that detailed kinetic analyses are needed.

Two amino acid positions have been described to be beneficial for C_4_ function by increasing the efficiency of PEPC by substitutions at position 890 and 780 (Blasing *et al.*, 2000; Paulus *et al.*, 2013). Studies on PEPC in *Flaveria* show that substitution of arginine with glycine at position 890 reduces the affinity for malate and aspartate which are inhibitors of PEPC. Studies also show that substitution of alanine by a serine at position 780 lowers the affinity (raises the *K*_m_) for PEP, meaning it would take higher levels of PEP to saturate the enzyme, which allows for higher concentrations of PEP to accumulate (Blasing *et al.*, 2000). Neither one of these sites was identified to be under positive selection in Suaedoideae (Table 1). Both *Schoberia* Kranz species sampled along with the two *Bienertia* species had a serine at position 780 (Supplementary Table S4 at *JXB* online). While none of the C_4_ species sampled had a glycine at residue 890, all the *Salsinia* Kranz C_4_ species as well as three C_3_ species had a methionine at this position (Supplementary Table S4). Conversely, there was positive selection for residues 880, 868, and 863, in order of proximity to 890, respectively (Fig. 3). These residues are close to the proposed site of inhibition by C_4_ acids aspartate and malate, with a substitution at residues 868 and 880 being present in the majority but not all C_4_ species (Supplementary Table S4). This lack of parallel amino acid conversion in Suaedoideae C_4_ species indicates either that these substitutions are not necessary for effective function of C_4_ due to compensating adjustments in C_4_ biochemistry, or growth conditions, or that alternative amino acid substitutions can fulfil these functions. These results also suggest that C_4_ may be able to function effectively with minimal changes in orthologous PEPC genes.

The results from variations in the branch-site test (Table 1), labelling all the Suaedoideae C_4_ branches or just the branches where C_4_ is thought to evolve, suggests there may be an order of selection or stronger selective pressures on residues at position 733 and 868 as noted above. At residue 733, all 12 C_4_ species had a substitution for something other than phenylalanine. However, as noted above, there is not strong selection for serine at position 780 (lacking in eight C_4_ species). This raises the question of how selective pressures on separate PEPC gene lineages may change if one mutation can affect the selective pressure on subsequent mutations in the same functional area of the enzyme. For example, if residue 733 mutates from the bulky phenylalanine to a less bulky amino acid (potentially increasing the affinity for HCO_3_^–^), before a substitution at 780 to serine (which may decrease the affinity for PEP), does this lower the selective pressure for a subsequent substitution at position 780? Reciprocally, would a substitution at residue 780 to serine lower the selective pressure for fixation of a mutation at residue 733, possibly explaining what is observed in *F. trinervia*, *Alternanthera pungens*, and *Mollugo cerviana* where a substitution at residue 733 is absent but there is a serine at residue 780? Thus, kinetic analysis for the different PEPC forms to determine how positive selection at specific codons affects the kinetic properties and response to allosteric effectors compared with the C_3_ orthologues is needed.

The substitution of serine for alanine at position 780 has been observed in almost every C_4_ species analysed to date (Blasing *et al.*, 2000; Christin *et al.*, 2007, 2011; Besnard *et al.*, 2009; Christin and Besnard, 2009). This serine substitution at position 780 has also been observed in the paralogous *ppc-B1* gene for the C_4_ species *Centropodia forskaliiis* (Christin *et al.*, 2007), although its not clear if this gene is being co-expressed with the *ppc-B2* gene or why there would be selection for this residue. However, *Hydrilla verticulata* (inducible aquatic single-cell C_4_ system) is an example of a PEPC gene that participates in C_4_ biochemistry but lacks the C_4_ signature serine—having the characteristic C_3_ alanine. Surprisingly, *in vitro* kinetic analysis showed that the serine was not essential for C_4_-like kinetics, and in fact substitution of serine for alanine in HVPEPC4 (the product of the PEPC gene that is proposed to function in the C_4_ cycle) was detrimental in terms of reduced *V*_max_ and *K*_cat_ values, although the same (serine for alanine) substitution in the anaplerotic form, HVPEPC3, altered the kinetics to become more C_4_ like (Rao *et al.*, 2008).

The results of the current study showing that only four out of 12 C_4_ species sampled (Table 2; Supplementary Table S4 at *JXB* online) have a serine at position 780, indicating that species can perform C_4_ photosynthesis with an alanine at position 780. *Bienertia sinuspersici* is the second C_4_ plant after *Mollugo cerviana* (*fragilis* group) that has been identified to have what appears to be a recent gene duplication of the *ppc-1* gene, with selection acting on only one of the two gene copies (Christin *et al.*, 2011). Whether both are expressed is not known, but if selection occurs on only one of the two paralogues it may shed light on regulation of C_4_ gene expression. Furthermore, *Mollugo cerviana* (*cerviana* group) populations that lacked a substitution for a serine residue at position 780 in the *ppc-1* gene were suggested not to be fully optimized for C_4_ biochemistry, although its carbon isotope composition is typical of c4 plants. Physiological analyses of species from the four C_4_ clades in Suaedoideae, which have diversity in the form of PEPC at position 780, indicate that they are functionally C_4_ (Smith *et al.*, 2009; King *et al.*, 2012). This indicates that the C4-ness of a species cannot be determined by Ser780 alone.

Finding C_4_ species without a Ser780 would not be the first time that experimental evidence for PEPC caused a re-evaluation of how enzyme kinetics are modified and the context of *in vivo* regulation. For example, PEPC has a conserved N-terminal serine that is subject to reversible phosphorylation in response to light and has been determined to reduce the inhibitory effects of malate *in vitro* (Tsuchida *et al.*, 2001). When this residue is not phosphorylated *in vivo*, there are no observable effects on CO_2_ assimilation rates in transgenic *F. bidentis*, raising the question that if phosphorylation is not essential for efficient C_4_ photosynthesis how biochemically is it related (Furumoto *et al.*, 2007). PEPCs from both single-cell C_4_ types and *S. eltonica* were shown to undergo phosphorylation in the light, at this conserved N-terminal serine analogous to other C_4_ systems, while the C_3_ plant *S. linifolia* did not (Lara *et al.*, 2006). This suggests there is the potential for tolerating accumulation of high levels of malate during photosynthesis in these C_4_ plants, but further analysis is needed to understand whether this is biochemically necessary and the context to *in vivo* levels of metabolites. This is analogous to the question of what effect substitution at position 780 in PEPC has on kinetics *in vitro*, and whether this can be observed to be beneficial under certain conditions *in vivo*.

## Conclusion

To date there are only a few reports on the recruitment of and modifications to PEPC along lineages that evolved C_4_ in the eudicots. In Suaedoideae, the *ppc-1* gene is used in C_4_ photosynthesis as observed in C_4_ eudicot families Amaranthaceae and Molluginaceae, which is analogous to the predominant recruitment of the same PEPC orthologue in C_4_ grasses and sedges. Unlike in the monocots, there is less evidence for the necessity for a high number of positively selected PEPC residues in Suaedoideae. Further analysis is needed to determine if the observed amino acid differences in Suaedoideae are more or less common across the Chenopodiaceae, their effect on PEPC kinetic properties, and ultimately how these changes are beneficial for C_4_ photosynthesis.

## Supplementary data

Supplementary data are available at *JXB* online.

Figure S1. Suaedoideae phylogeny, using only the *ppc1* third position plus intron sequence, that was used for positive selection analysis.

Figure S2. Cartoon representation of the C_4_-PEPC enzyme structure of *Flaveria trinervia* (*ppc-2* gene) (Paulus *et al.*, 2013), showing the spatial effect of a substitution at residue 733 (maize numbering) in relation to residue 606.

Table S1. Name, sequence of primer, and which species the primer was used for in sequencing Suaedoideae *ppc-1* and *ppc-2* genes.

Table S2. List of species origin, voucher, and *ppc* sequence accession numbers generated in this study.

Table S3. Chenopodioideae species list used in phylogenetic analyses with marker accession numbers.

Table S4. Comparison of *ppc-1* exon 8, 9, and 10 amino acids, that were identified to be under positive selection in Suaedoideae, across Eudicot families.

Supplementary Data

## Abbreviations:

dN: non-synonmous substitution rate
dS: synonymous substitution rate
G6P: glucose 6-phosphate
LRT: likelihood ratio test
ML: maximum likelihood
OAA: oxaloacetate
PEP: phosphoenolpyruvate
PEPC: phosphoenolpyruvate carboxylase
ω: the non-synonymous/synonymous substitution rate ratio.
