# Supplementary material for: Positive selection of Kranz and non-Kranz C4 phosphoenolpyruvate carboxylase amino acids in Suaedoideae (Chenopodiaceae)
Source: J Exp Bot. 2014 Mar 5;65(13):3595–607. doi: 10.1093/jxb/eru053 (PMC4085955; doi:10.1093/jxb/eru053)
Supplement: Supplementary Data [file supp_65_13_3595__index.html]

Positive selection of Kranz and non-Kranz C4 phosphoenolpyruvate carboxylase amino acids in Suaedoideae (Chenopodiaceae) — Positive selection of Kranz and non-Kranz C4 phosphoenolpyruvate carboxylase amino acids in Suaedoideae (Chenopodiaceae) — Supplementary Data 

# Positive selection of Kranz and non-Kranz C4 phosphoenolpyruvate carboxylase amino acids in Suaedoideae (Chenopodiaceae)

## Supplementary Data

Data files

**Files in this Data Supplement:**

- Supplementary Data - Supplementary Data
