# Supplementary material for: Positive selection of Kranz and non-Kranz C4 phosphoenolpyruvate carboxylase amino acids in Suaedoideae (Chenopodiaceae)
Source: J Exp Bot. 2014 Mar 5;65(13):3595–607. doi: 10.1093/jxb/eru053 (PMC4085955; doi:10.1093/jxb/eru053)
Supplement: Supplementary Data [file supp_eru053_jexbot114330_file001.pdf]

Evolutionary Dynamics of phosphoenolpyruvate carboxylase in Kranz and non-Kranz C4 Suaedoideae (Chenopodiaceae)

Josh J. Rosnow · Gerald E. Edwards · Eric H. Roalson

Supplemental Table 1. Name, sequence of primer, and which species the primer was used for in sequencing Suaedoideae *ppc-1* and *ppc-2* genes.

Primer Name	Primer Sequence 5' --> 3'	Species *ppc-1* genes sequenced using this primer
SP6	ATTTAGGTGACACTATAG	All
T7	TAATACGACTCACTATAGGG	All
PEPCA-Ex8-430FW	GGTAAGGATGCTGGTCGTCT	S. acuminata, S. aegyptiaca, S. altissima, S. aralocaspica, S. eltonica, S. fruticosa, S. heterophylla, S. moquinii, S. nigra, S. occientalis, S. taxifolia, S. vera, S. vermiculata
PEPCA-Ex8-430RV	AGACGACCAGCATCCTTACC	S. acuminata, S. aegyptiaca, S. altissima, S. aralocaspica, S. eltonica, S. fruticosa, S. heterophylla, S. moquinii, S. nigra, S. occientalis, S. taxifolia, S. vermiculata
PEPCA-5010FW	ATTGCAGCGGTATACAGCTG	S. calceoliformis, S. linearis, S. maritima, S. occientalis
PEPCA-5010RV	CAGCTGTATACCGCTGCAAT	S. acuminata, S. aegyptiaca, S. altissima, S. aralocaspica, S. calceoliformis, S. eltonica, S. fruticosa, S. heterophylla, S. linearis, S. linifolia, S. maritima, S. moquinii, S. nigra, S. occientalis, S. taxifolia, S. vera, S. vermiculata
PEPC-Ex9-1246RV	GGCCACTCATTGTACATCTC	S. acuminata, S. aegyptiaca, S.aralocaspica, S.eltonica, S. fruticosa, S. heterophylla, S. linearis, S. linifolia, S. maritima, S. moquinii, S. nigra, S. occientalis, S. taxifolia, S. vera, S. vermiculata
PEPCA-Ex9-1062FW	GAGTTRGAGTATGGGCGC	S. acuminata, S. aegyptiaca, S. aralocaspica, S. eltonica, S. fruticosa, S. heterophylla, S. linearis, S linifolia, S. maritima, S. moquinii, S. nigra, S. occientalis, S. taxifolia, S. vermiculata
PEPCA-Ex10-730FW	CAGGTTGCTGGGCACAAA	S. acuminata, S. aegyptiaca, S. altissima, S. aralocaspica, S. eltonica, S. fruticosa, S. heterophylla, S. linifolia, S. moquinii, S. nigra, S. occientalis, S. taxifolia, S. vera, S.vermiculata
PEPCA-Ex10-730RV	TTTGTGCCCAGCAACCTG	S. acuminata, S. aegyptiaca, S. altissima, S. aralocaspica, S. eltonica, S. fruticosa, S. heterophylla, S. linifolia, S. moquinii, S. nigra, S. occientalis, S. physophora, S. taxifolia, S. vera, S. vermiculata
PEPCA-5000FW	TGGCTTGGGTTTGGAGCAG	S. acuminata, B. cycloptera, Sa. divracata, S. eltonica, Sa. gesneroides, S. linifolia, S. maritima, S. physophora, B, sinuspersici, S. vera
PEPCA-5000RV	CTGCTCCAAACCCAAGCCA	B. cycloptera, Sa. divracata, Sa. gesneroides, S. maritima, S. physophora, B. sinuspersici
Aegy_Ex9II_FW	ATTAYGAAGAAACTCTGAAG	S.aegyptiaca, S. moquinii, S. nigra, S. taxifolia, S.vermiculata
Aegy_Ex9AA_FW	TTATCAGAATATGTAATATGCATCT	S. vermiculata
Aegy_Ex9AA_RV	AGATGCATATTACATATTCTGATAA	S. aegyptiaca, S. moquinii, S. vera, S. vermiculata
PEPCA-LinEx9FW	CAGGTTCCATTTGCCTGTGT	S. altissima, S. linearis
PEPCA-LinEx9RV	ACACAGGCAAATGGAACCTG	S. altissima, S. linearis,
PEPC FW 4230	TTTGAGAAACTTGCTGATCTSGAG	S. calceoliformis, B. cycloptera, S. linearis, S. physophora, B. sinuspersici
PEPCA-10-II-FW	AGAAACTTTTTYTCCAGGTA	S. calceoliformis, S. occientalis
PEPC-10A-II-RV	TACCTCTAAGAGAAGGTCCT	S. calceoliformis, S. occientalis
PEPC 4789 FW	CGATTTGTGGAGTAYTTCCG	S. calceoliformis, B. cycloptera, Sa. divracata, S. eltonica, Sa. gesneroides, S. occientalis, B. sinuspersici
PEPC FW 4057	TTGTTTGGYYCTGATCTCCCCAA	B. cycloptera, B. sinuspersici
PEPCAEx8-964-RV	GTGGCAGCAACAGCCAT	B. cycloptera, Sa. divracata, Sa. gesneroides, S. physophora, B. sinuspersici
PEPC 4354	CAAGAAGYTATGATYGGDTA	B. cycloptera
BieA_I9FW	AGGTATGTCTTTGCTTTACA	B. cycloptera, B. sinuspersici
BieA_I9RV	TGTAAAGCAAAGACATACCT	B. cycloptera, B. sinuspersici
GesA_E8_FW	GTGAAGCTTACCATGTTTCA	Sa. divracata, Sa. gesneroides, S. linifolia, S. vera
GesA_E8_RV	TGAAACATGGTAAGCTTCAC	Sa. divracata, Sa. gesneroides, S. vera
DivA_I9FW	AATAGATTTTAATACAAAGG	Sa. divracata
DivA_I9RV	CCTTTGTATTAAAATCTATT	Sa. divracata
GesA_I9_FW	TGAATACAAAGGTAAAACCGC	Sa. gesneroides
GesA_I9_RV	GCGGTTTTACCTTTGTATTCA	Sa. gesneroides
BreA_E8FW	TGATGGCAAGCAGGAAGTCAT	S. maritima, S. occientalis
PhyA_E10FW	CTTCGTGATCCTTACATCAC	S. maritima, S. physophora
PhyA_E10RV	GTGATGTAAGGATCACGAAG	S. maritima, S. physophora
PEPCA-Ex8-470RV	AGCTCCTCCTGAACTTTGTA	S. nigra, S. vera
P-Phys_I10_FW	GGTATCAGCGCTAGCGTAACA	S. physophora
P-Phys_I10_RV	TGTTACGCTAGCGCTGATACC	S. physophora
**Primer Name**	**Primer Sequence 5' --> 3'**	**Species *ppc-2* genes sequenced using this primer**
SP6	ATTTAGGTGACACTATAG	All
T7	TAATACGACTCACTATAGGG	All
PEPC FW 4057	TTGTTTGGYYCTGATCTCCCCAA	S. accuminata, S. aegyptiaca, S. altissima, S. aralocaspica, S. eltonica, S. fruticosa, S. heterophylla, S. maritima, S. moquinii, S. physophora, S. taxifolia, S. vermiculata
PEPC 4354	CAAGAAGYTATGATYGGDTA	S. accuminata, S. aegyptiaca, S. altissima, S. aralocaspica, S. eltonica, S. fruticosa, S. heterophylla, S. maritima, S. moquinii, S. physophora, S. vermiculata
PEPC 4789 FW	CGATTTGTGGAGTAYTTCCG	S. accuminata, S. aegyptiaca, S. altissima, S. aralocaspica, S. eltonica, S. fruticosa, S. heterophylla, S. maritima, S. moquinii, S. physophora, S. taxifolia, S. vermiculata
PEPCIntron10A-FW	AGAAACTTCTTCTCCAGGTA	S. accuminata, S. aegyptiaca, S. altissima, S. aralocaspica, S. eltonica, S. fruticosa, S. heterophylla, S. maritima, S. moquinii, S. physophora, S. taxifolia, S. vermiculata
PEPCIntron10A-RV	TACCTGGAGAAGAAGTTTCT	S. accuminata, S. aegyptiaca, S. altissima, S. aralocaspica, S. eltonica, S. fruticosa, S. heterophylla, S. maritima, S. moquinii, S. physophora, S. taxifolia, S. vermiculata
Hetero-b-RV	CTTTGAAAACAATRGAACGAT	S. heterophylla
Elton-b-RV	GTCTGTTTGACAAAT	S. accuminata, S. eltonica
PEPC4D-RV	AGAGTGGCAGCAGTGAAACG	S. accuminata
Aral-b-RV	AAATCATTCAAATATTTATT	S. aralocaspica
Fruit-b-RV	ATGCCAGTCATTAATTTAT	S. fruticosa
Moqin-b-RV	TCTGATCTTTAGTTATTAAAA	S. moquinii

Supplementary Table 2. List of species origin, voucher, and *ppc* sequence accession numbers generated in this study. Number in parentheses refers to the isolate number.

Species	Origin	Voucher	*ppc-1*	*ppc-1* (2nd gene)	*ppc-2*
Bienertia cycloptera	Kavir Protected Area near Mobarakiyeh, Iran	WS386420	KF964064 (A1)	KF964065 (A16)	-
Bienertia sinuspersici	Kuwait, collected by Abdulrahman Alsirhan	WS386421	KF964066 (UK1)	KF964067 (A9)	-
Suaeda acuminata	Armenia, collected by Maria Lomonosova	WS386425	KF964068 (A1)	KF964069 (A2)	KF964105 (B6)
Suaeda aegyptiaca	Kew, 011776	WS369795	KF964070 (A1)	KF964071 (A5)	KF964106 (B1)
Suaeda altissima	Armenia, collected by Maria Lomonosova	WS386426	KF964072 (A2)	KF964073 (A7)	KF964107 (B16)
Suaeda aralocaspica	Kazakhstan	WS369789	KF964074 (A2)	KF964075 (A3)	KF964108 (B3)
Suaeda calceoliformis	USA, Nevada, from H. Freitag	WS386422	KF964076 (A10)	KF964077 (A4)	-
Suaeda eltonica	Western Kazakhstan	WS369797	KF964078 (A2)	KF964079 (A3)	KF964109 (B15)
Suaeda fruticosa	Pakistan	WS386427	KF964080 (A2)	KF964081 (A5)	KF964110 (B9)
Suaeda heterophylla	United States,	WS369803	KF964082 (A1)	KF964083 (A2)	KF964111 (B10)
Suaeda linearis	USA, New Jersey, from H. Freitag	WS386424	KF964084 (A7)	KF964085 (A1)	-
Suaeda linifolia	United States	WS369788	KF964086 (A1)	KF964087 (A15)	-
Suaeda maritima	Kew, 59651	WS369798	KF964088 (A19)	KF964089 (A20)	KF964112 (B4)
Suaeda moquinii	Kew 0204473	WS386429	KF964090 (A1)	KF964091 (A3)	KF964113 (B1)
Suaeda nigra	GRIN, W6 27337	WS386428	KF964092 (A1)	KF964093 (A3)	-
Suaeda occientalis	USA, Nevada, from H. Freitag	WS386423	KF964094 (A1)	KF964095 (A1A)	-
Suaeda physophora	Lake Elton, Soljanka river. Russia	WS386430	KF964096 (A7)	-	KF964114 (B14)
Suaeda taxifolia	United States, California	WS369802	KF964097 (A3)	KF964098 (A1)	KF964115 (B3)
Suaeda vera	Kew, 0083962	WS386431	KF964099 (A1)	KF964100 (A3)	-
Suaeda vermiculata	Kew, 0118549	WS369796	KF964101 (A1)	KF964102 (A3)	KF964116 (B3)
Salsola divaricata	Canary Islends, coll. H. Akhani	WS386546	KF964103 (A1)	-	-
Salsola genistoides	Spain	WS386547	KF964104 (A1)	-	-

Supplementary Table 3: Chenopodioideae species list used in phylogenetic analyses with marker accession numbers.

Species	Reference	ITS-5.8S	atpB-rbcL	psbB-psbT-psbN
Salicornioideae				
Kalidium caspicum Ung.-Sternb.	Kapralov et al. (2006)	DQ499339	DQ340097	DQ499423
Salicornia europaea L.	Schutze et al. (2003)	AY181941	AY181814	AY181941
Salsoloideae				
Salsola canescens (Moq.) Boiss.	Kapralov et al. (2006)	DQ499346	DQ499369	DQ499430
Salsola kali L.	Pyankov et al. (2001)	AF318646	DQ499370	DQ499431
Suaedoideae				
Brezia				
S. australis (R.Br.) Moq.	Schutze et al. (2003)	AY181826	AY181766	AY181891
S. calceoliformis (Hooker) Moq.	Kapralov et al. (2006)	DQ499351	DQ499376	DQ499436
S. corniculata (C.A.Mey.) Bunge	Schutze et al. (2003)	AY181841	AY181780	AY181905
S. crassifolia Pall.	Schutze et al. (2003)	AY181820	AY181760	AY181885
S. heterophylla (Kar. & Kir.) *Bunge*	Schutze et al. (2003)	AY181837	AY181776	AY181901
S. maritima (L.) Dumort.	Schutze et al. (2003)	AY181818	AY181758	AY181883
S. kossinskyi Iljin,	Kapralov et al. (2006)	DQ499352	DQ499377	DQ499437
S. occidentalis (S.Watson) S.Watson	Kapralov et al. (2006)	DQ499353	-	DQ499438
S. pannonica (Beck) Graebn.	Schutze et al. (2003)	AY181839	AY181778	AY181903
S. aff. *patagonica* Speg.	Schutze et al. (2003)	AY181843	AY181782	AY181907
S. prostrata Pall.	Schutze et al. (2003)	AY181834	AY181773	AY181898
S. spicata (Willd.) Moq.	Schutze et al. (2003)	AY181828	AY181767	AY181892
S. tschujensis Lomonosova & Freitag	Schutze et al. (2003)	AY181838	AY181777	AY181902
Schanginia				
S. linifolia Pall.	Schutze et al. (2003)	DQ499357	AY181805	AY181932
S. paradoxa Bunge	Schutze et al. (2003)	AY181871	AY181806	AY181933
Borszczowia				
S. aralocaspica (Bunge) Freitag & Schutze	Kapralov et al. (2006)	DQ499350	DQ499374	DQ499435
Physophora				
S. physophora Pall.	Schutze et al. (2003)	DQ499356	AY181802	-
S. ifniensis Caball.	Schutze et al. (2003)	AY181866	AY181800	AY181928
S. palaestina Eig. & Zohary	Schutze et al. (2003)	AY181865	AY181799	AY181927
Salsina				
S. aegyptiaca (Hasselq.) Zohary	Schutze et al. (2003)	AY181853	AY181788	AY181917
S. divaricata Moq.	Schutze et al. (2003)	AY181863	AY181797	AY181926
S. foliosa Moq.	Schutze et al. (2003)	AY181862	AY181796	AY181925
S. nigra (Raf.) J.F.Macbride	Kapralov et al. (2006)	FJ449800	-	DQ499439
S. taxifolia (Standley) Standley	Kapralov et al. (2006)	DQ499354	DQ499378	DQ499440
S. altissima (L.) Pall.	Schutze et al. (2003)	AY181850	AY181785	AY181914
S. arcuata Bunge	Schutze et al. (2003)	AY181854	AY181789	AY181918
S. articulata Aellen	Schutze et al. (2003)	AY181860	AY181795	AY181924
S. asphaltica Boiss.	Schutze et al. (2003)	AY181851	AY181786	AY181915
S. dendroides (C.A.Mey.) Moq.	Schutze et al. (2003)	AY181856	AY181791	AY181920
S. fruticosa Forssk. & J.F.Gmelin	Schutze et al. (2003)	DQ499355	AY181793	AY181922
S. microphylla Pall.	Schutze et al. (2003)	AY181855	AY181790	AY181919
S. moquinii (Torr.) Greene	Schutze et al. (2003)	AY181864	AY181798	-
S. monoica Forssk. & J.F.Gmelin	Schutze et al. (2003)	AY181859	AY181794	AY181923
S. vermiculata Forssk. & J.F.Gmelin	Schutze et al. (2003)	AY181852	AY181787	AY181916
Schoberia				
S. acuminata (C.A.Mey.) Moq.	Schutze et al. (2003)	DQ499358	FJ449757	AY181912
S. carnosissima Post	Schutze et al. (2003)	AY181846	AY181783	AY181910
S. cucullata Aellen	Schutze et al. (2003)	AY181845	FJ449760	AY181909
S. eltonica Iljin	Schutze et al. (2003)	AY181847	AY181784	AY181911
S. microsperma (C.A.Mey.) Fenzl	Kapralov et al. (2006)	DQ499359	DQ499375	DQ499442
S. splendens (Pourr.) Gren. & Godr.	Schutze et al. (2003)	AY181844	FJ449781	AY181908
Alexandra				
S. lehmannii Bunge	Kapralov et al. (2006)	DQ499347	DQ499371	DQ499432
Suaeda				
S. vera Forssk. & J.F.Gmelin	Schutze et al. (2003)	AY181868	AY181803	AY181930
S. ekimii	Schutze et al. (2003)	AY181869	AY181804	AY181931
Bienertia				
B. cycloptera Bunge	Kapralov et al. (2006)	DQ499348	DQ499372	DQ499433
B. sinuspersici Akhani,	Kapralov et al. (2006)	DQ499349	DQ499373	DQ499434

Supplemental Table 4. Comparison of *ppc-1* exon 8, 9 & 10 amino acids, that were identified to be under positive selection in Suaedoideae, across Eudicot families. The type of photosynthesis is listed after each species name. The number in parentheses indicates the number of species sampled. The fraction represents the number of species that have the noted substitution (amino acids on the right of the arrow). All amino acids shown were identified to be under positive selection at the P>0.95 interval, except residue 733 which was at the P>0.99 interval. * indicates amino acids identified to be under positive selection by Christin et al. (2007) at the P>0.95. ^ indicates amino acids identified to be under positive selection by Besnard et al. (2009) at the P>0.999. Amino acids numbering is based on *Zea mays* PEPC (CAA33317).

	480 (D→E)	485 (K→A)	513 (D→A)	519 (H→K)	627 (V→I)	662 (D→E)	695 (T→V/I)	707 (I→M/L/S/T)	733 ^* (F→M/L/R)	735 (E→N)	744 (L→C/R)	780 [x] (A→S)	794 * (F→L/I/M)	863 * (S→N/K/D/T)	868 (K→R/Q/L)	880 (D→N/Y/E)	890 (R→G)	931 (M→I)
**Suaedoideae**																		
Bienertia cycloptera - non-Kranz C_4	D	K	D	H	V	D	I	I	R	E	L	S	F	N	K	D	R	I
Bienertia sinuspersci - non-Kranz C_4 (FSW allele)	E	K	A	H	I	D	V	M	R	E	L	A	F	N	K	D	R	I
Bienertia sinuspersci - non-Kranz C_4 (FAW allele)	E	K	D	H	I	D	V	M	R	E	L	S	F	N	K	D	R	M
Suaeda aralocaspica - non-Kranz C_4	E	K	A	H	I	D	T	S	L	E	L	A	I	T	Q	E	R	M
Schoberia - Kranz C_4 (2)	2/2 E	K	0/2	2/2 K	0/2	0/2	0/2	2/2 L	2/2 M	0/2	2/2 C	2/2 S	1/2 L	2/2 N	2/2 R	0/2	0/2	0/2
Salsinia - Kranz C_4 (7)	7/7 E	7/7 A	7/7 A	0/7	7/7 I	3/7	0/8	1/7 T	7/7 L	7/7 N	2/7 R	0/7	7/7	7/7	7/7 L	6/7	0/7	0/7
Salsola diviricata - C_3-C_4 intermediate	w	K	D	H	V	D	T	I	F	E	L	A	F	S	K	D	R	M
Salsola gesnoridies - C_3	D	K	D	H	V	D	T	I	F	E	L	A	F	S	K	D	R	M
Suaeda - C_3 (8)	0/8	0/8	0/8	0/8	0/8	0/8	0/8	0/8	0/8	0/8	0/8	0/8	0/8	0/8	0/8	0/8	0/8	0/8
**Amaranthaceae**																		
Amaranthus hypochondriacus - Kranz C_4	S	K	D	R	V	D	T	T	L	E	S	S	C	S	Q	D	I	M
Alternanthera pungens - Kranz C_4	A	K	D	H	I	G	T	S	F	E	R	S	F	S	Q	D	R	M
Alternanthera sessilis - C_3-C_4 intermediate	D	K	D	H	V	D	T	S	F	E	L	A	F	S	K	D	R	M
Alternanthera ficoides - C_3	D	K	D	H	V	D	T	S	F	E	L	A	F	S	K	D	R	M
**Molluginaceae**																		
Mollugo cerviana - Kranz C_4 (FSW allele)	E	K	D	R	I	D	T	L	F	E	L	S	I	N	K	D	R	M
Mollugo verticillata - C_3-C_4 intermediate	E	K	D	H	V	D	T	I	F	E	L	A	F	N	K	D	R	M
Mollugo molluginis - C_3	E	K	D	H	V	D	T	I	F	E	L	A	F	N	K	D	R	M
**Brassicaceae *s. l.***																		
Cleome gynandra - Kranz C_4	E	K	D	H	L	D	T	I	L	E	L	S	I	N	K	D	M	M
Arabidopsis thaliana - C_3	E	K	D	K	V	D	T	V	F	E	L	A	F	N	R	D	R	M
**Asteraceae (*ppc-2*)**																		
Flaveria trinervia - Kranz C_4	E	K	D	K	I	D	C	I	F	E	L	S	F	N	K	D	G	A
Flaveria brownii - C_4-like	E	K	D	K	V	D	C	I	F	E	L	A	F	N	K	D	R	A
Flaveria pringlei - C_3	E	K	D	K	V	D	C	I	F	E	L	A	F	N	K	D	R	A
**Monocot (*ppc-B2*)**																		
Zea maize - C_4	E	K	D	H	V	D	T	V	V	E	S	S	V	K	Q	D	G	A

Supplemental Figure 1. Suaedoideae phylogeny, using only *ppc1* third position plus intron sequence, that was used for positive selection analysis. Numbers above branches refer to bootstrap percentages. Abbreviations: *B.* = *Bienertia*, *S.* = *Suaeda*.

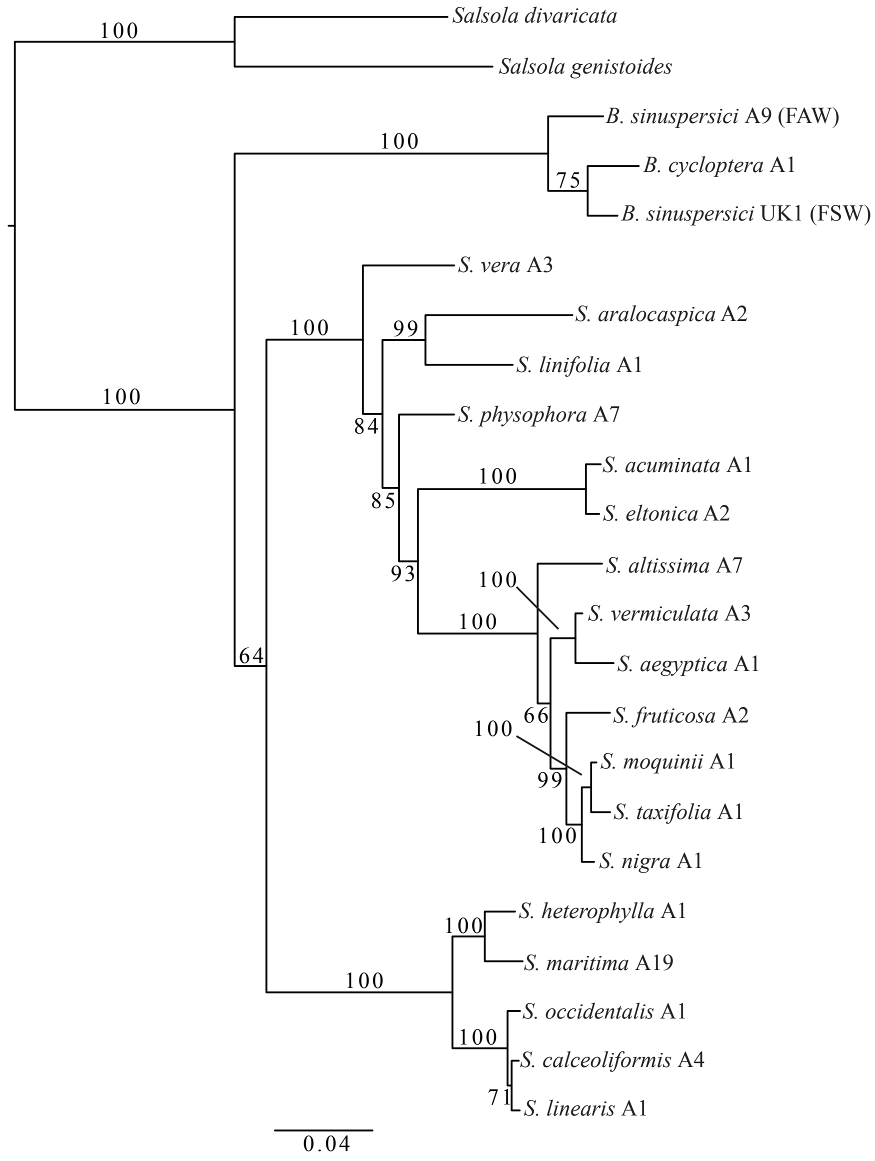

Supplemental Figure 2. Cartoon representation of the C_4-PEPC enzyme structure of *Flaveria trinervia* (*ppc-2* gene) (Paulus et al. 2013), showing the spatial effect of a substitution at residue 733 (maize numbering) in relation to residue 606. A) Basal enzyme state (C_3) with phenylaalaine present at residue 733. B) Substitution at residue 733 for leucine (the most prevalent substitution). C) Substitution at residue 733 for methionine. D) Substitution at residue 733 for arginine. Green residues are sites under positive selection, biochemically essential residues are in a shade of red (histone 177 is bright red, Mg^{2+} binding are ruby, PEP and HCO_3^- binding are dark red, lysine 606 is brown), malate/aspartate allosteric regulatory sites are light blue, and serine residue 780 is yellow.

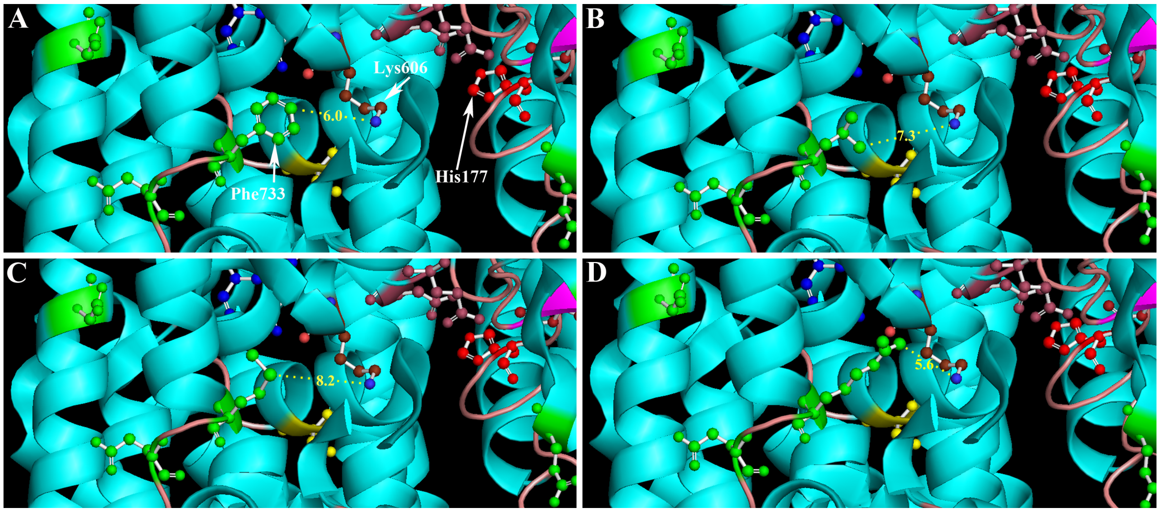

References:

Besnard G, Muasya AM, Russier F, Roalson EH, Salamin N, Christin P-A. 2009. Phylogenomics of C_4 photosynthesis in sedges (Cyperaceae): multiple appearances and genetic convergence. *Molecular Biology and Evolution* **26**, 1909-1919.

Christin P-A, Salamin N, Savolainen V, Duvall MR, Besnard G. 2007. C_4 photosynthesis evolved in grasses via parallel adaptive genetic changes. *Current Biology* **17**, 1241-1247.

Kapralov MV, Akhani H, Voznesenskaya EV, Edwards G, Franceschi V, Roalson EH. 2006. Phylogenetic relationships in the Salicornioideae / Suaedoideae / Salsoloideae s.l. (Chenopodiaceae) clade and a clarification of the phylogenetic position of *Bienertia* and *Alexandra* using multiple DNA sequence datasets. *Systematic Botany* **31**, 571-585.

Paulus JK, Schlieper D, Groth G. 2013. Greater efficiency of photosynthetic carbon fixation due to single amino-acid substitution. *Nature Communications* **4**.

Pyankov VI, Artyusheva EG, Edwards GE, Black Jr. CC, Soltis PS. 2001. Phylogenetic analysis of tribe Salsoleae (Chenopodiaceae) based on ribosomal ITS sequences: impications for the evolution of photosynthetic types. *American Journal of Botany* **88**, 1189-1198.

Schutze P, Freitag H, Weising K. 2003. An integrated molecular and morphological study of the subfamily Suaedoideae Ulbr. (Chenopodiaceae) *Plant Systematics and Evolution* **239**, 257-286.
